# One Hundred Consecutive Cases of Skin Disease, II

**Published:** 1891-02

**Authors:** M. B. Hutchins

**Affiliations:** Lecturer on Diseases of the Skin, Atlanta Medical College


### ONE HUNDRED CONSECUTIVE CASES OF SKIN DISEASE.

II.

By M. B. HUTCHINS, M. D.,
Lecturer on Diseases of the Skin, Atlanta Medical College.

The case of eczema of eyelids was that of a delicate girl of ten years. Present all her life. Crusts and scales at roots of lashes, matting them together. Lids of right eye most affected, the upper the worse. Conjunctivæ healthy-looking, but a little pus in inner canthus. Ordered to cleanse eyelids with warm water, and apply the "regulation,"

℞. Hydrarg. ox. flav., gr. ss.
Ungt. petrolat., ʒii.

M.

so commonly prescribed for the affections of margins of lids. I do not know the result in this case, and it is chiefly of interest because a dermatologist would call it eczema and an oculist, probably, blepharitis marginalis, and each treat it the same way. The child was also given the syrup of iodide of iron.

The four cases of eczema of the face were as follows:

First.—A medical student, aged twenty-eight. Hands slightly affected, showing a general tendency to the disease. Skin over nose and malar eminences thickened, with "oozing points," and a few fissures. Bowels constipated. This relieved by the mixture of cascara sagrada, tincture of nux vomica, and water—which has proven of permanent benefit when taken in doses graded according to effect. Following is "the average:"

℞. Casc. sagradæ, ℥i.

Tinct. nucis vom., ʒi.

Aquæ, ad. ℥iij.

M. Sig.—ʒi after meals.

Locally:

℞. Picis liquidæ,

Zinci oxidi, aa ℥ii.

Ungt. simp., ℥ii.

M.

Applied, and avoidance of application of water enjoined. Skin was nearly normal in ten days; then patient got a very imperfectly prepared ointment and finally, after a relapse, left the city, only "improved." Relapses are frequent in eczema, and sometimes apparently without cause, but careful investigation will generally bring to light some irregularity which explains the occurrence.

Second of face, woman of forty-five. Eczema similar to above, but situated in a rosaceic skin, redness and dilatation of vessels appearing as if chronic. Improved under treatment similar to case first, and then moved away from the city. Internally she took—

℞. Ferri et quiniæ cit., ʒi.

Liq. potass. ars., ʒiss.

Tinct. cinchon. comp., ℥ii.

Aqua, ad. ℥iv.

Mix. Sig.—℥i after meals.

"Fowler's solution" was given in a few of these chronic, inactive cases, but I could not decide as to its being of especial value as an adjuvant to local treatment.

Third of face, infant of ten months. Face swollen, crusted, excoriated, and discharging profusely a sero-purulent fluid. About base of thumbs a few irregular pustules. Similar condition on legs. These attributed to simple pus-infection. Ordered water kept from face; apply—

℞. Ac. sal., gr. x.

Zinc. ox., ʒi.

Ungt. simp., ℥ij.

M.

Sig.—Apply thoroughly and place cloth mask over face. Rub a little on legs and hands. Much improved in a week, and finished treatment, I heard, with an ointment of "zinc and diachylon," previously in the house.

Fourth, of face, boy of eight, situated on cheeks and about orbits. Only scaly papules; surrounding skin normal in color. With a lens could be seen in the centre of these lesions a pin-point sized clear vesicle, perfect in shape. Patient delicate in appearance, and there was one enlarged lymphatic gland on back of neck. Ten drops of syrup of iodide of iron were given after meals, and the ointment of salicylic acid and zinc oxide applied. Eruption and glandular enlargement had disappeared at end of a week.

First case of eczema of hands, woman of fifty-one. Duration, "better or worse," about thirty-five years. Skin on backs of hands and fingers, rough, thickened, stiff; fissures here and there. Tips of some of the fingers showed the parchment-feeling, raised epidermis, so characteristic of eczema in this situation. Palms but slightly affected, nearly normal. Condition of skin aggravated by use of hands in housework, and handling salt articles, etc., in a grocery store kept by herself. Her grandfather and two or three other relatives had similar condition. It is likely that there was an element of ichthyosis—or "fish-skin"—in the family, or else a very strong eczematous tendency. General health good, save constipation. The "cascara and nux" mixture relieved this. Salicylic and zinc oxide proper strength, with ungt. diachyli first employed, bandaged on and rubber gloves directed worn to protect hands. Fissures healed in a week. In two weeks nothing save a little thickening and scaling remained, and the palms, though about as at first, "looked worse" than the backs of the hands. Could not induce patient to protect hands thoroughly, and she finally abandoned treatment.

Second case, that of mechanic, aged twenty-eight. Present nearly a year. Location, adjacent sides, index and middle fingers of right hand, and over metacarpo-phalangeal junction. Skin thickened, scaly, tending to fissure. Treated by binding on ungt. picis et zinc oxidi, renewing morning and evening; protection from water. After progressive improvement for two weeks, the bandages and salve were displaced by the "pigment" of liquid pitch, ether and alcohol, as described in preceding article.

Was discharged in less than two months, practically well. The slight relapses which he had were treated with the ointment, and the treatment concluded with the "pigment." This pigment is invaluable to "finish up" a case in which only slight scaling and thickening remain.

There was, also, the case of a negro woman seen at the college in February of last year. Skin of palms very hard, thickened and deep fissures in natural lines of the skin. Under the use of diachylon ointment and wearing rubber gloves, the condition rapidly improved in ten days, after which the patient was not seen.

One case of eczema of the fingers, dentist of fifty-eight. Excited by accidental cauterization of fingers. Ends of thumbs and fingers affected, the latter especially about nails. In places only thickening and scaling ; in others fissuring also. Deep, clear vesicles in places in palmar skin, as of that obstructed condition of sweat glands usually called "pompholyx." The "salicylic and oxide of zinc ointment" produced quite rapid improvement in eczematous condition. The "pompholyx" condition was relieved by substitution of the following for ointment :

R. Resorcini, gr. xx.

Zinci oxidi, ʒii.

M. Amyli ,ad. ℥i.

This being bandaged on or worn in cloth glove fingers. But the two conditions present made the case just so much more difficult to manage, the drying effect of the powder tending to cause cracking of the skin, while the use of the ointment (and rubber covering sometimes employed) seemed to favor formation of vesicles. The case finally passed from my care in this unsatisfactory, though improved, condition.

These "hand cases" present great difficulty in treatment, just because it is impossible to get the average patient to give the requisite rest and protection to the hands.

(*Ordinary Eczema, concluded next issue.*)

16½ Whitehall St.

